# *Rtfc (4931414P19Rik*) Regulates *in vitro* Thyroid Differentiation and *in vivo* Thyroid Function

**DOI:** 10.1038/srep43396

**Published:** 2017-02-23

**Authors:** Yang Yu, Chang Liu, Junxia Zhang, Mimi Zhang, Wei Wen, Xianhui Ruan, Dapeng Li, Shuang Zhang, Ming Gao, Lingyi Chen

**Affiliations:** 1 Department of Thyroid and Neck Tumor, Tianjin Medical University Cancer Institute and Hospital, National Clinical Research Center for Cancer, Key Laboratory of Cancer Prevention and Therapy, Tianjin, Huanhuxi Road, Ti-Yuan-Bei, Hexi District, Tianjin 300060, China; 2 State Key Laboratory of Medicinal Chemical Biology, Key Laboratory of Bioactive Materials, Ministry of Education, Tianjin Key Laboratory of Protein Sciences and College of Life Sciences, Nankai University, Tianjin 300071, China; 3 Tianjin Women’s and Children’s Health Center, Tianjin 300070, China; 4 State Key Laboratory of Molecular Oncology, Cancer Institute/Hospital, Chinese Academy of Medical Sciences, Beijing 100021, China

## Abstract

Thyroid is a one of the most important endocrine organs. Understanding the molecular mechanism underlying thyroid development and function, as well as thyroid diseases, is beneficial for the clinical treatment of thyroid diseases and tumors. Through genetic linkage analysis and exome sequencing, we previously identified an uncharacterized gene *C14orf93 (RTFC*, mouse homolog: *4931414P19Rik*) as a novel susceptibility gene for familial non-medullary thyroid carcinoma, and demonstrated its function in promoting thyroid tumor. However, the role of *RTFC* in thyroid development and function remains unexplored. In this study, we found that knockout of *Rtfc* compromises the *in vitro* thyroid differentiation of mouse embryonic stem cells. In contrast, *Rtfc*^−/−^ mice are viable and fertile, and the size and the morphology of thyroid are not affected by *Rtfc* knockout. However, female *Rtfc*^−/−^ mice, but not male *Rtfc*^−/−^ mice, display mild hypothyroidism. In summary, our data suggest the roles of *Rtfc* in *in vitro* thyroid differentiation of embryonic stem cells, and *in vivo* thyroid function.

Thyroid is one of the most important endocrine organs in vertebrate. It plays important roles in development, growth, and metabolism through secreting thyroid hormones^1^,^2^. Thyroid hormones regulate many developmental processes, including neural, bone, and reproductive organ development^3^,^4^,^5^. It is well known that lack of sufficient thyroid hormone in early human development stages results in cretinism. In adults, the primary effects of thyroid hormones are regulating metabolism such as protein, carbohydrate, lipid, and vitamin metabolism^6^.

Thyroid is the first emerging endocrine organ during embryogenesis, formed around the 22nd day post conception in humans^7^. Onset of thyroid hormone synthesis represents the accomplishment of thyroid development^8^. Thyroid transcription factors (TTFs), including NK2 homeobox 1 (NKX2-1), forkhead box protein E1 (FOXE1), paired box protein 8 (PAX8) and haematopoietically expressed homeobox (HHEX), are expressed in thyroid precursor cells, and also important for the functional differentiation of the gland in late development and postnatally^9^,^10^,^11^,^12^,^13^,^14^. NKX2-1, FOXE1 and PAX8 cooperate to activate genes that drive thyroid hormone synthesis, such as *TG, TPO*, and *SLC5a5*^15^,^16^,^17^. Given the importance of TTFs in development, cell proliferation, and differentiation, mutations in these genes are associated with thyroid disorders. For example, mutations in *NKX2-1, FOXE1* and *PAX8*, as well as polymorphisms in *FOXE1*, are associated with thyroid dysgenesis^18^,^19^,^20^,^21^,^22^,^23^,^24^,^25^,^26^. Moreover, genetic alterations of *NKX2-1, PAX8*, and *FOXE1*, have been reported to be related to thyroid cancer^27^,^28^,^29^,^30^.

Through genetic linkage analysis and exome sequencing, we previously identified *C14orf93* (also named as Regulator of Thyroid Function and Cancer, *RTFC*) as a novel susceptibility gene for familial nonmedullary thyroid cancer (FNMTC)^31^. The oncogenic functions of R115Q, V205M, and G209D RTFC mutants are demonstrated by cell surviving assay, migration assay, and colony forming assays. Moreover, RTFC has been identified as a potential antigen associated with the pathogenesis of peripheral T-cell lymphomas, not otherwise specified (PTCL, NOS)^32^. Two *in vitro* biochemical screens suggested that RTFC might have RNA and phosphopeptide (pSer/pThr-X-X-X-pSer/pThr) binding activities^33^,^34^. Yet, the role of *RTFC* in normal development, as well as the molecular function of RTFC, remain unexplored.

In this study, we investigated the function of *Rtfc* (originally named *4931414P19Rik*) in mouse thyroid development and thyroid function. We found that knockout of *Rtfc* compromises the thyroid differentiation of mouse embryonic stem cells (ESCs), while overexpression of *RTFC* promotes mouse ESCs to differentiate toward the thyroid lineage. In contrast, *Rtfc*^−/−^ mice are viable and fertile, and the size and the morphology of thyroid are not affected by *Rtfc* knockout. However, female *Rtfc*^−/−^ mice, but not male *Rtfc*^−/−^ mice, display mild hypothyroidism. In summary, our data demonstrated the roles of *RTFC* in *in vitro* thyroid differentiation of ESCs, and *in vivo* thyroid function.

## Results

### *Rtfc* is involved in thyroid differentiation of mouse ESCs

*Rtfc* expression dynamics during human and mouse development were looked up from NCBI UniGene EST profiles (https://www.ncbi.nlm.nih.gov/unigene/) and EMBL-EBI database (http://www.ebi.ac.uk/), respectively (Fig. S1). It is notable that *Rtfc* is expressed at low levels during embryo development, and its expression increases and hits the peak at the juvenile stage, implying a functional role of *Rtfc* in development.

**Figure 1:**
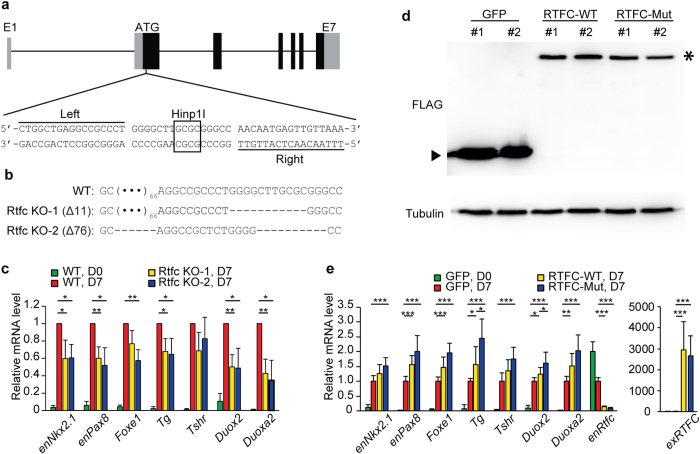
*Rtfc* is involved in thyroid differentiation of mouse ESCs. (**a**) The design of TALENs to knock out the *Rtfc* gene. Boxes represent exons of the *Rtfc* gene. The coding regions are shown in black. (**b**) Sequences at the TALENs targeting site in two *Rtfc* knockout (KO) clones. (**c**) Knockout of *Rtfc* reduces the thyroid differentiation efficiency of mouse ESCs. WT ESCs and two *Rtfc* KO ESC lines were differentiated toward the thyroid lineage. The expression levels of thyroid markers were measured in day 7 differentiated cells (D7) by quantitative RT-PCR. Day 0 undifferentiated WT ESCs (WT, D0) were included as a control to show the activation of thyroid markers after differentiation. Data are shown as mean ± SD (n = 3). (**d**) Western blot to detect the overexpression of FLAG tagged GFP, WT and V205M RTFC proteins in mouse ESCs. Stable mouse ESC lines overexpressing FLAG tagged GFP, WT and V205M RTFC were established and subjected to Western blot. A triangle indicates the band of FLAG-GFP, and an asterisk marks the band of FLAG-RTFC. Cropped blots are shown, and full-length blots are presented in Supplementary Figure S5. (**e**) Overexpression of WT and V205M RTFC promote the thyroid differentiation of mouse ESCs. ESCs overexpressing GFP, FLAG tagged WT and V205M RTFC, were differentiated toward the thyroid lineage. The expression of thyroid markers in day 7 differentiated cells (D7), as well as in day 0 undifferentiated GFP ESCs (GFP, D0), were analyzed. Data are shown as mean ± SD (n = 6, including 2 independent clones and 3 technical replicates).

To investigate the role of *Rtfc* in the normal development of thyroid, we took advantage of an *in vitro* thyroid differentiation system of mouse ESCs by overexpression of *Nkx2.1* and *Pax8*^35^. An “*Nkx2.1*-IRES-*Pax8*” cassette driven by a tetracycline-inducible promoter was integrated into the engineered *ColA1* locus through FLPe recombinase–mediated recombination in KH2 ESCs, resulting in a Dox-inducible *Nkx2.1* and *Pax8* ESC line (iN/P ESCs). When iN/P ESCs were induced to differentiate into thyroid cells, the expression level of *Rtfc* slightly increases, similar to its expression dynamics during *in vivo* embryogenesis (Fig. S2). A pair of transcription activator-like effector nucleases (TALENs) were designed and constructed to disrupt the *Rtfc* gene in iN/P ESCs (Fig. 1a). Two *Rtfc* knockout iN/P ESC lines were established, and sequencing of the TALENs targeting sites validated the disruption of *Rtfc* alleles (Fig. 1b). When *Rtfc* knockout and WT iN/P ESCs were differentiated toward the thyroid lineage, the up-regulation of endogenous thyroid genes, including *Nkx2.1, Pax8, Foxe1, Tg, Duox2*, and *Duoxa2*, are reduced in *Rtfc* knockout cells, compared to WT cells (Fig. 1c). Conversely, overexpression of human WT and V205M (a mutation identified in a FNMTC pedigree^31^) *RTFC* enhance the activation of thyroid genes during ESC differentiation (Fig. 1d and e). These data suggest that *Rtfc* contributes to *in vitro* thyroid differentiation of ESCs. Moreover, V205M RTFC mutant appears to be more potent in promoting thyroid differentiation (Fig. 1e), implying that this mutation enhances the function of RTFC, consistent with our previous data that V205M increases the colony forming capacity of thyroid cancer cells^31^.

### *Rtfc* ^−/−^ mice are viable and fertile

**Figure 2:**
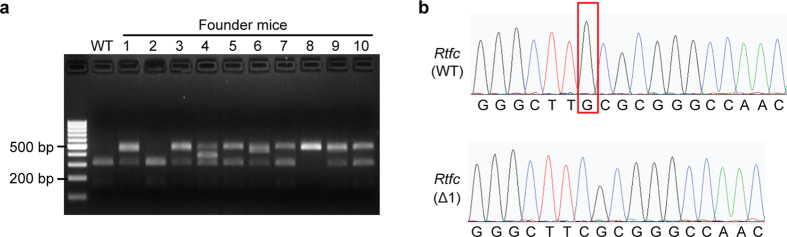
Generation of *Rtfc* knockout mice. (**a**) Genotyping of the founder mice. Only one allele of *Rtfc* was modified in founder mice 1, 3, 5, 6, 7, 9 and 10, while both *Rtfc* alleles were modified in founder mouse 8. It appears that there were three *Rtfc* alleles in founder mouse 4. Neither *Rtfc* allele was modified in founder mouse 2. (**b**) Sanger sequencing result of the representative homozygous knockout mouse reveals a 1-bp deletion in two *Rtfc* gene alleles. The red box marks the deleted G in the *Rtfc*^−/−^ (Δ1) mouse.

To further demonstrate the function of *Rtfc* in thyroid development *in vivo*, one *Rtfc* homozygous and eight *Rtfc* heterozygous knockout founder mice were generated by zygotic injection of TALEN mRNA (Fig. 2a). Sequencing result showed a 1-bp deletion (Δ1) of the *Rtfc* gene in the *Rtfc* homozygous knockout (*Rtfc*^−/−^) founder mouse (Fig. 2b). And this founder mouse was used for subsequent mating and experiments. Mating between female and male *Rtfc*^+/*−*^ mice yielded *Rtfc*^+/+^, *Rtfc*^+/*−*^, and *Rtfc*^*−/−*^ progenies, and the ratio of three genotypes is close to the expected Mendelian ratio of 1:2:1 (Table 1). Moreover, live pups were born by *Rtfc*^*−/−*^ parents. Thus, *Rtfc*^*−/−*^ mice are viable and fertile.

### Female *Rtfc* ^−/−^ mice display mild hypothyroidism

**Figure 3:**
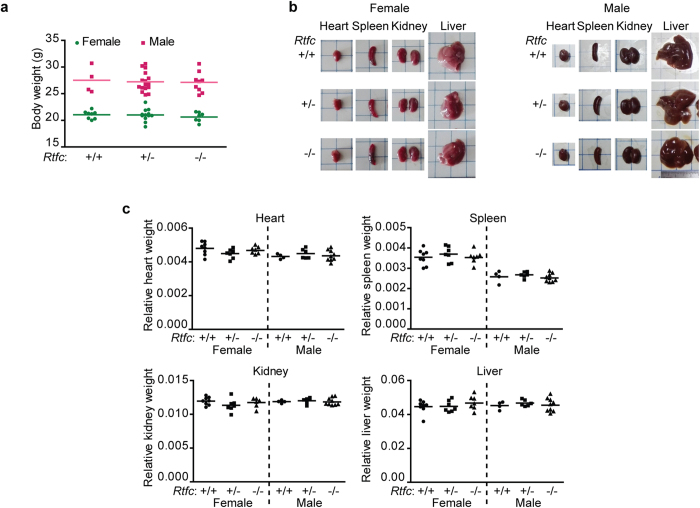
Phenotypic analysis of *Rtfc* knockout mice. (**a**) Body weight of mouse is not affected by *Rtfc* knockout. (**b**) Images of heart, spleen, kidney and liver, demonstrate that the size and the morphology of these organs are similar in *Rtfc*^+/+^, *Rtfc*^+/*−*^ *and Rtfc*^−/−^ mice. (**c**) *Rtfc* knockout does not affect the relative weight of heart, spleen, kidney and liver (normalized to the body weight).

**Figure 4:**
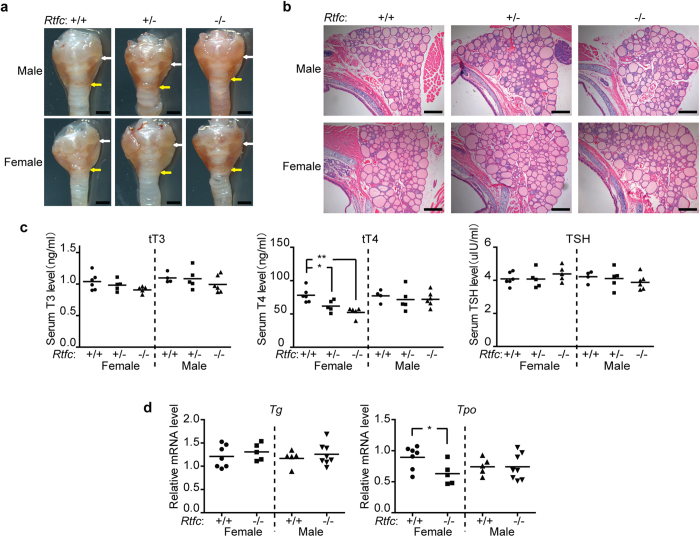
Female mice display mild hypothyroidism. (**a**) Thyroid morphology of representative 3-month old *Rtfc*^+/+^, *Rtfc*^+/*−*^ and *Rtfc*^−/−^ male and female mice. White and yellow arrows indicate the upper and lower edges of thyroid, respectively. Scale bars: 1 mm. (**b**) HE staining of thyroid tissue sections of 3-month old *Rtfc*^+/+^, *Rtfc*^+/*−*^ and *Rtfc*^−/−^ male and female mice. Scale bars: 200 μm. (**c**) Serum total T3, total T4 and TSH levels of 3-month old *Rtfc*^+/+^, *Rtfc*^+/*−*^ and *Rtfc*^−/−^ male and female mice, measured by radioimmunoassay. Each dot represents the value in a mouse, and bars are averages. (**d**) Expression of *Tg* and *Tpo* in the thyroid of 3-month old *Rtfc*^+/+^ and *Rtfc*^−/−^ male and female mice. Each dot represents the value in a mouse, and bars are averages.

Next, we examined the weight and morphology of liver, spleen, heart, kidney, as well as body weight of *Rtfc*^*−/−*^ mice, and found them indistinguishable from those of *Rtfc*^+/+^ and *Rtfc*^+/*−*^ mice (Fig. 3a–c). In addition, no difference in thyroid size and morphology was observed among *Rtfc*^+/+^, *Rtfc*^+/*−*^, and *Rtfc*^*−/−*^ mice of both genders (Fig. 4a). Tissue section and HE staining revealed normal follicle size and structure in the thyroid of *Rtfc*^*−/−*^ mice (Fig. 4b). Furthermore, knockout of *Rtfc* does not affect the expression of seven thyroid-related genes (*Nkx2-1, Pax8, Foxe1, Tshr, Duox2, Duoxa2* and *Nis*) in the thyroid (Fig. S3).

As the size and the morphology of thyroid are not affected by *Rtfc* knockout, we further tested the function of thyroid, by measuring the levels of total T3, total T4 and TSH in serum. Knockout of *Rtfc* does not affect the serum T3, T4, and TSH levels in male mice. Rather, it reduces the serum T4 level only in female mice, and the serum T4 level in female *Rtfc*^*−/−*^ mice is the lowest (Fig. 4c). Compared with female *Rtfc*^+/+^ mice, the serum T3 and TSH levels of female *Rtfc*^*−/−*^ mice slightly decreases and increases, respectively, even though the differences are statistically insignificant (Fig. 4c). These data suggest that *Rtfc* knockout compromises the function of thyroid and results in mild hypothyroidism in female mice, but not in male mice.

To understand how thyroid function is compromised in female *Rtfc*^−/−^ mice, we examined the expression of two critical genes *Tg* and *Tpo* for the production of thyroid hormones, in the thyroid of female *Rtfc*^−/−^ and *Rtfc*^+/+^ mice. *Tg* encodes thyroglobulin, which is used by thyroid to produce the thyroid hormones T3 and T4. *Tpo* encodes thyroperoxidase, which catalyzes both the iodination of tyrosine residues and the coupling of iodotyrosine residues in thyroglobulin, for the production of T3 and T4^36^. Our results showed that the expression of *Tpo*, but not *Tg*, is reduced in female *Rtfc*^−/−^ mice, in comparison to female *Rtfc*^+/+^ mice (Fig. 4d). Thus, the reduced level of *Tpo* in the thyroid of *Rtfc*^−/−^ female mice, might account for the decrease of serum T4. Consistent with the unchanged serum T3, T4 and TSH levels in *Rtfc*^−/−^ male mice, the expression levels of *Tg* and *Tpo* are not affected by *Rtfc* knockout in male mice (Fig. 4d). Nevertheless, it remains possible that other factors, such as a pituitary effect of Rtfc, contribute to the decreased thyroid hormones and the relatively steady level of TSH.

## Discussion

Previously, we have demonstrated the role of *RTFC* in thyroid cancer^31^. In this study, we investigated the role of *Rtfc* in the development of thyroid. Knockout of *Rtfc* reduces the thyroid differentiation efficiency of mouse ESCs, suggesting the role of *Rtfc* in thyroid development. However, *Rtfc*^−/−^ mice are viable and fertile, and the size and the morphology of thyroid in *Rtfc*^−/−^ mice are indistinguishable from WT mice. This discrepancy might be due to the more permissive and optimized environment for thyroid development *in vivo*. When ESCs differentiate into the thyroid lineage *in vitro*, some extrinsic cues, such as growth factors and signaling molecules, might be missing or compromised. Thus, the effect of *Rtfc* KO on thyroid can only be detected in the *in vitro* ESC differentiation system. Nevertheless, female *Rtfc*^−/−^ mice, but not male *Rtfc*^−/−^ mice, display mild hypothyroidism. These data validate the *in vivo* role of *Rtfc* in regulating the function of thyroid. Lack of any thyroid phenotype in male *Rtfc*^−/−^ mice might be due to a more robust regulatory mechanism in male, thus compensating the loss of *Rtfc*. Consistently, thyroid diseases are more prevalent in women than in men, which might be caused by gonadal hormones and/or genes encoded by sexual chromosomes^37^,^38^. Even though only mild thyroid defects were identified in female *Rtfc*^−/−^ mice, it remains possible that *Rtfc* knockout might have subtle effects in other tissues, which requires further investigation.

The molecular function of RTFC remains elusive. *RTFC* is widely expressed in a variety of tissues in both mouse and human (Fig. S4) and the RTFC proteins are highly conserved among different species, suggesting the functional importance of the *RTFC* gene. For example, human and mouse RTFC proteins share 87.5% of identity. Through analyzing the protein sequence, no structural domain is identified, except for a putative signal peptide at the N-terminus of RTFC. However, a pilot study to characterize the subcellular distribution of human proteins reveals that RTFC is mainly localized in the nucleus and the membrane^39^. It is interesting that overexpression of *RTFC* in mouse ESCs suppresses endogenous *Rtfc* expression (Fig. 1e), suggesting that Rtfc protein may function as a transcriptional regulator, negatively regulating the transcription of its own gene. Whether RTFC is secreted remains to be proved. In addition, RTFC has been identified in two screens for RNA binding proteins and for phosphopeptide binding proteins^33^,^34^. However, these biochemical properties of RTFC have not been further validated and linked to any biological process.

In summary, our data revealed the biological function of *Rtfc* in regulating the development and the function of thyroid. The role of Rtfc in other organs and the molecular functions of Rtfc require further investigation.

## Methods

### Ethics statement

All animal experiments were carried out in strict accordance with the recommendations in the Guide for the Care and Use of Laboratory Animals of the National Institutes of Health. The use of mice for this research is approved by Nankai Animal Care and Use Committee (Approval number: 20140001).

### Cell culture

KH2 mouse ESCs were cultured in growth medium consisting of 85% DMEM (high glucose, Invitrogen), 15% fetal bovine serum (FBS, Hyclone), 2 mM L-glutamine, 5000 units/ml penicillin and streptomycin, 0.1 mM nonessential amino acids (Invitrogen), 0.1 mM 2-mercaptoethanol (Sigma), and 1000 units/ml LIF (ESGRO, Chemicon).

### Construction of cell lines

Inducible *Nkx2.1*-IRES-*Pax8* (iN/P) ESCs were constructed from the parent cell line, KH2. In brief, the *Nkx2.1*-IRES-*Pax8* cassette was inserted into the flp-in vector pgkATGfrt. The resulting vector and an Flpe expression vector were co-electroporated into KH2 cells. Twenty-four hours after electroporation, the cells were selected with 140 μg/ml hygromycin (Roche) for 10 days. The remaining hygromycin resistant colonies were individually picked. The correct recombination events were verified by genomic DNA PCR. Quantitative RT-PCR was also carried out to confirm the induced expression of *Nkx2.1* and *Pax8* upon doxycycline (Dox, 5 μg/ml) treatment.

A pair of TALEN vectors targeting the exon 2 of mouse *Rtfc* gene (Fig. 1a) were constructed. Recognition sequences of TALENs are 5′-CTGGCTGAGGCCGCCCT-3′ (left arm) and 5′-TTTAACAACTCATTGTT-3′ (right arm). Mouse ESCs were transfected with two TALEN vectors using Lipofectamine™ 2000 (Invitrogen). Forty-eight hours after transfection, transfected ESCs were re-seeded in a 6-well plate at 500 cells per well. After 5–8 days, individual colonies were picked. Disruption of the HinP1I site in the *Rtfc* locus was first screened by PCR amplification and HinP1I (NEB) digestion. Then sequencing analysis was carried out to confirm the frame-shift mutations in mutated ESC clones.

To construct stable *GFP*, WT and V205M *RTFC* overexpression iN/P ESCs, plasmids overexpressing *GFP*, WT, and V205M *RTFC* were transfected into iN/P ESCs using Lipofectamine™ 3000 (Invitrogen). Twenty-four hours after transfection, the transfected cells were selected with 500 μg/ml G418 (Gibco) for 9 days. The remaining G418 resistant colonies were individually picked. The integration of exogenous DNA was verified by genomic DNA PCR. Quantitative RT-PCR and Western blot were also carried out to validate the overexpression of WT and V205M *RTFC*.

### Thyroid differentiation of ESCs

Embryoid bodies (EBs) were formed by culturing ESCs in hanging drops (1, 000 cells per 25 μl drop) in EB differentiation medium (mouse ESC growth medium without LIF, but supplemented with 50 μg/ml ascorbic acid (Sigma)). On day 4, EBs were harvested and plated in gelatin-coated 6-well plates (20 EBs/well) in EB differentiation medium supplemented with 5 μg/ml Dox (Sigma) for 3 days to allow further differentiation.

### Generation of *Rtfc* knockout mice

The same TALENs were used to knockout *Rtfc* in mouse ESCs and in mice. TALEN mRNAs were transcribed *in vitro* using the Sp6 mMESSAGE mMACHINE Kit (Ambion). TALEN mRNAs (10 ng/μl) were injected into the cytoplasma of fertilized eggs of C57BL/6 mice. The injected embryos were cultured in KSOM with amino acids at 37 °C under 5% CO2 in air for 24 hours, and then transferred into pseudopregnant mice. One-cell embryo injection and embryo transfer were carried out by Cyagen biosciences (Guangzhou, China).

### Genotyping of *Rtfc* alleles

Genomic DNA was extracted from mice toes or cells using phenol-chloroform method, and PCR amplification was performed with primers, 5′-TGGAGCACTTGTGAAGCGACCTAT-3′ and 5′-AGTTGCTGGCTTCTCCTGGACTT-3′. PCR products were then digested by HinP1I at 37 °C. DNA fragments were separated in 2% agarose gel by electrophoresis. For WT *Rtfc* allele, the PCR products were completely digested into two fragments of 304 bp and 162 bp. For mutated *Rtfc* allele, the amplified DNA cannot be cut by HinP1I, resulting in a single ~466 bp band.

### Western Blot Analysis

Cells were lysed in lysis buffer (Beyotime), and protein concentration was measured using a BCA protein assay kit (Beyotime) to ensure equal loading. The samples were resolved by SDS-PAGE, followed by transferring onto a PVDF membrane (Millipore). Membranes were probed with anti-C14orf93 (Sigma), anti-FLAG (Sigma), anti-tubulin (Huada, Beijing). Bound primary antibodies were recognized by HRP-linked secondary antibodies (GE Healthcare). Immunoreactivity was detected by ECL Plus (Beyotime). Digital images were taken by the automatic chemiluminescence imaging analysis system (Tanon).

### Quantitative RT–PCR

Total RNA was extracted from cells using RNeasy Mini kit (Qiagen). cDNA synthesis was performed using Transcriptor First Strand cDNA Synthesis kit (Roche) according to manufacturer’s instruction. PCR reactions were performed with FastStart Universal SYBR Green Master (Roche) in a BioRad iQ5 system. PCR cycling conditions were: 95 °C for 10 min, 40 cycles of 95 °C for 15 s, 58 °C for 15 s, and 72 °C for 30 s, and then a melting curve of the amplified DNA was acquired. Quantification of target genes was normalized with β-Actin. Primer information was listed in Supplementary Table S1.

### Thyroid dissection

Three-month old mice were sacrificed by cervical dislocation, and then thyroids were taken out by dissecting of necks. Images were acquired with Leica M165 FC Fluorescent Stereo Microscope.

### Hematoxylin and eosin (HE) staining

Dissected thyroids were fixed using 4% PFA at 4 °C for 24 h, dehydrated through xylenes and alcohols, and embedded in paraffin. Sections were cut at 5 μm and stained with haematoxylin and eosin for histological examination. Images were taken with Leica DM3000 Microscope.

### Measurement of serum thyroid hormones and TSH

Serum total thyroxine (T4), total 3,3′,5-Triiodothyronine (T3) and thyroid stimulating hormone (TSH) were measured with Iodine [^125^I] Thyroxine Radioimmunoassay Kit, Iodine [^125^I] 3,3′,5-Triiodothyronine Radioimmunoassay Kit, and Iodine [^125^I] Human Thyroid Stimulating Hormone Radioimmunoassay Kit (Beijing north institute of biological technology, Beijing), respectively. These radioimmunoassays were performed by Beijing Kemeixinyun Biotechnology Company.

### Statistical analysis

All data were analyzed by Student’s t-test. Statistically significant p values were indicated in figures as follows: ***p < 0.001, **p < 0.01, *p < 0.05. Additional information of methods is provided in online supplementary file.

## Additional Information

**How to cite this article:** Yu, Y. *et al. Rtfc (4931414P19Rik)* Regulates *in vitro* Thyroid Differentiation and *in vivo* Thyroid Function. *Sci. Rep.* **7**, 43396; doi: 10.1038/srep43396 (2017).

**Publisher's note:** Springer Nature remains neutral with regard to jurisdictional claims in published maps and institutional affiliations.

## Supplementary Material

Supplementary Information

## Figures and Tables

**Table 1:** Mating between *Rtfc* ^+/*−*^ mice demonstrates that *Rtfc* ^−/−^ mice are viable.

	*Rtfc*^−/−^	*Rtfc*^+/*−*^	*Rtfc*^+/+^	Total
Strain 1 (Δ41)	11 (28.9%)	15 (39.5%)	12 (31.6%)	38
Strain 2 (Δ1)	51 (23.0%)	126 (56.7%)	45 (20.3%)	222
