# Supplementary material for: Rtfc (4931414P19Rik) Regulates in vitro Thyroid Differentiation and in vivo Thyroid Function
Source: Sci Rep. 2017 Feb 23;7:43396. doi: 10.1038/srep43396 (PMC5322522; doi:10.1038/srep43396)
Supplement: Supplementary Information [file srep43396-s1.pdf]

Supplementary Information

Rtfc (*4931414P19Rik*) Regulates *in vitro* Thyroid Differentiation and *in vivo* Thyroid Function

Yang Yu [1, 5], Chang Liu [2, 5], Junxia Zhang [2], Mimi Zhang [2], Wei Wen [2], Xianhui Ruan [1], Dapeng Li [1], Shuang Zhang [3], Ming Gao [1, *], Lingyi Chen [2, 4, *]

[1] Department of Thyroid and Neck Tumor, Tianjin Medical University Cancer Institute and Hospital, National Clinical Research Center for Cancer, Key Laboratory of Cancer Prevention and Therapy, Tianjin, Huanhuxi Road, Ti-Yuan-Bei, Hexi District, Tianjin 300060, China

[2] State Key Laboratory of Medicinal Chemical Biology, Key Laboratory of Bioactive Materials, Ministry of Education, Tianjin Key Laboratory of Protein Sciences and College of Life Sciences, Nankai University, Tianjin 300071, China

[3] Tianjin Women's and Children's Health Center, Tianjin 300070, China

[4] State Key Laboratory of Molecular Oncology, Cancer Institute/Hospital, Chinese Academy of Medical Sciences, Beijing 100021, China

[5] Co-first authors

[*] Correspondence: gaoming68@aliyun.com (M.G.), lingyichen@nankai.edu.cn (L.C.)

Running Title: *Role of Rtfc in thyroid development and function*

Supplementary Figures and Legends

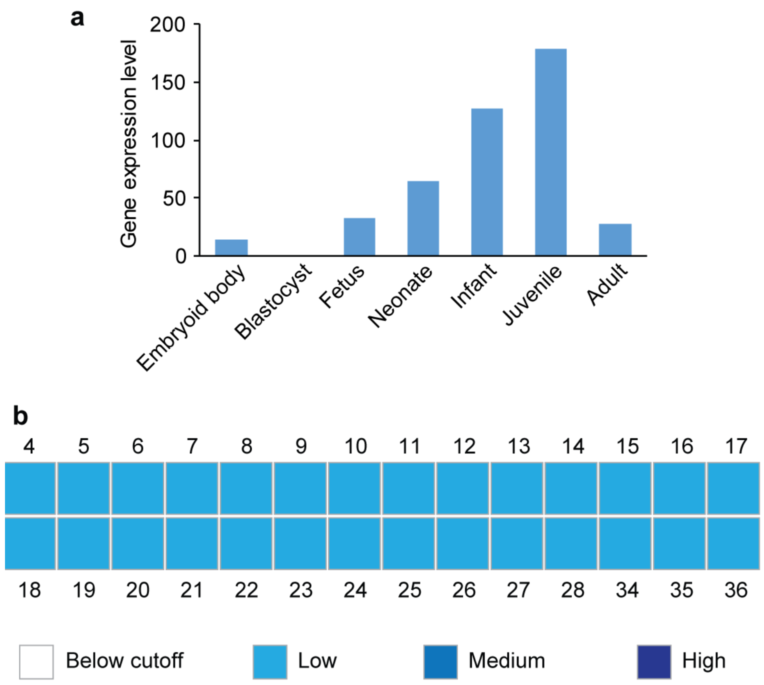

Figure S1. *RTFC* (*Rtfc*) expression during human and mouse development.

(a) *RTFC* expression levels at various developmental stages of human. Data is extracted from NCBI EST profiles (https://www.ncbi.nlm.nih.gov/unigene/). The values shown in the y-axis are transcripts per million (TPM). (b) *Rtfc* is expressed, but at a low level, during mouse embryo development. Data is extracted from EMBL-EBI database (http://www.ebi.ac.uk/). The numbers above or below squares indicate the corresponding somite stages.

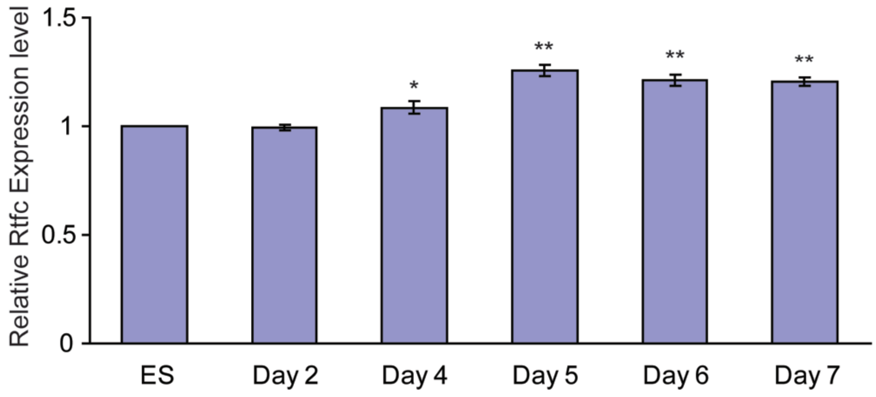

Figure S2. *Rtfc* expression during ESC differentiation toward the thyroid lineage.

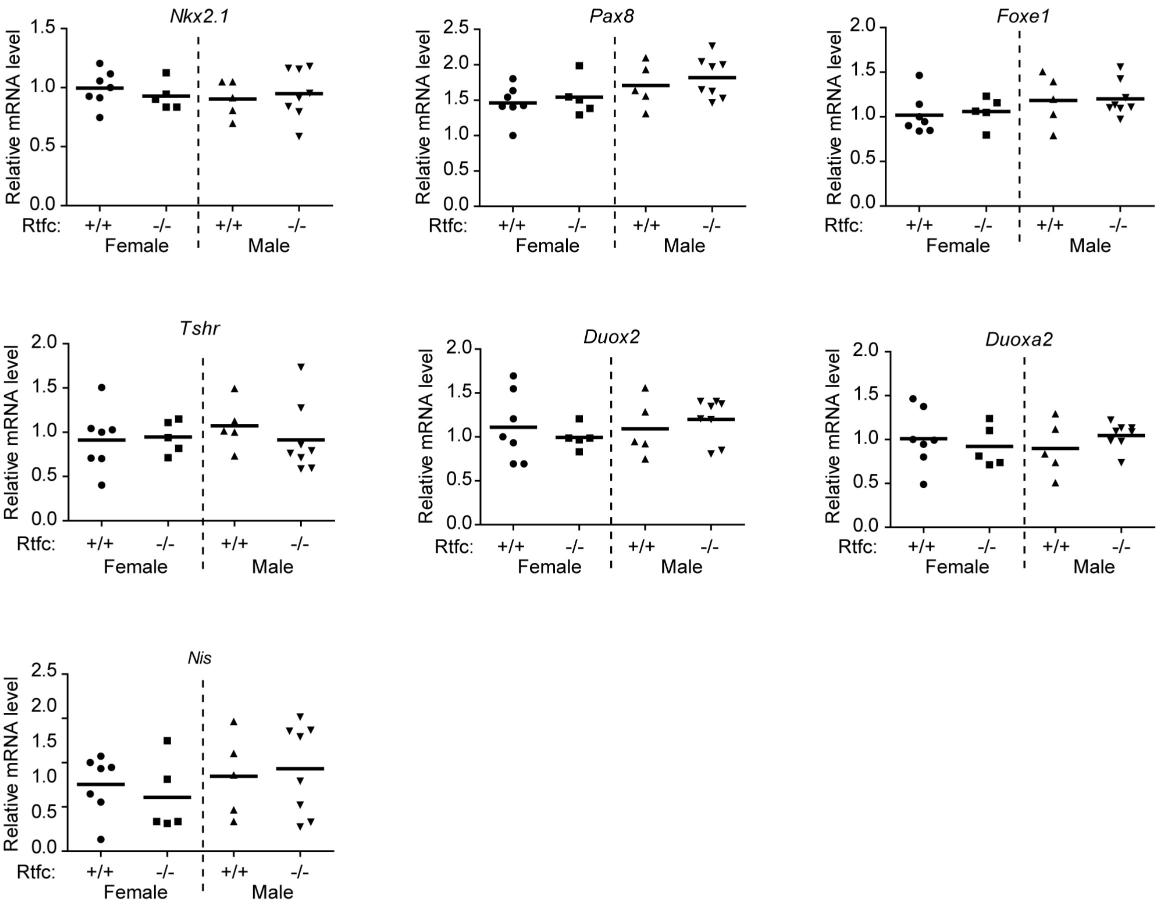

Figure S3. Knockout of *Rtfc* does not affect the expression of most thyroid-related genes. Expression of *Nkx2-1*, *Pax8*, *Foxe1*, *Tshr*, *Duox2*, *Duoxa2* and *Nis* in the thyroid of 3-month old *Rtfc*$^{+/+}$ and *Rtfc*$^{-/-}$, male and female mice, were examined by quantitative RT-PCR. Each dot represents the expression level in a mouse, and bars are averages.

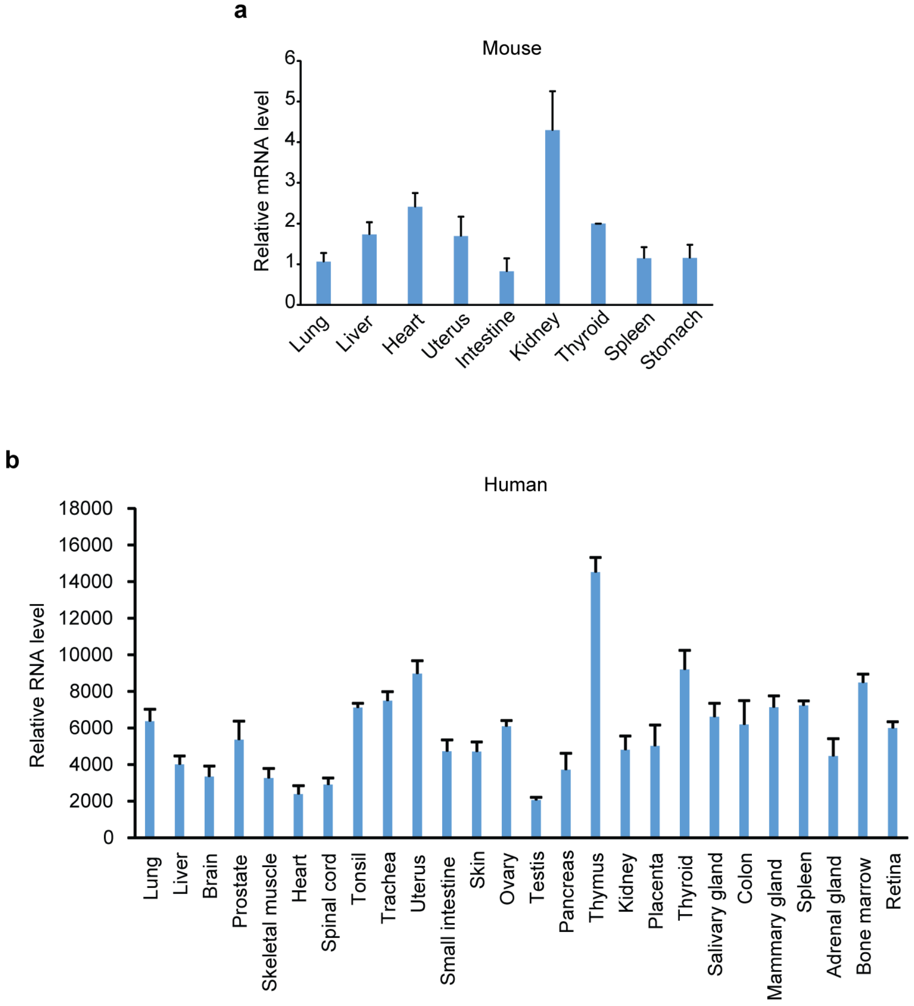

Figure S4. *RTFC* (*Rtfc*) expression in mouse and human tissues.

(a) *Rtfc* mRNA level in various mouse tissues. Total RNA isolated from mouse lung, liver, heart, uterus, intestine, kidney, thyroid, spleen and stomach, was subjected to quantitative RT-PCR. *β-Actin* was used as an internal control. (b) *RTFC* mRNA expression level in various normal human tissues. The microarray data is extracted from GEO database (GDS3113), and the gene expression level is the average value of three samples in each tissue.

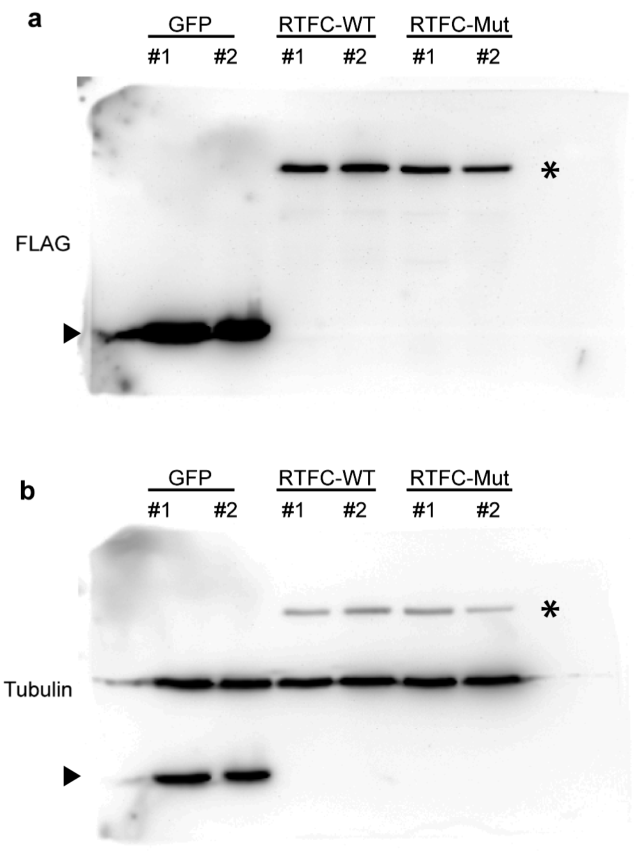

Figure S5. Full-length blots of the cropped blots in Figure 1d.

(a) The blot was probed with anti-FLAG antibody to detect FLAG tagged GFP (marked with a triangle) and RTFC (marked with an asterisk). (b) The blot shown in (a) was stripped, and re-probed with anti-Tubulin antibody. Some signals from previous detection (marked with a triangle and an asterisk) were retained due to incomplete stripping.

Supplementary Table

Table S1. Primers used for quantitative RT-PCR.

Gene name	Forward primer	Reverse primer
RTFC	ACCAGTCCCCACAATTACAC	CAGGTCCAAAATGCCTCATG
β-Actin	CAGAAGGAGATTACTGCTCTGGCT	TACTCCTGCTTGCTGATCCACATC
enNkx2.1	GGCGCCATGTCTTGTTCT	GGGCTCAAGCGCATCTCA
enPax8	CAGCCTGCTGAGTTCTCCAT	CTGTCTCAGGCCAAGTCCTC
Foxe1	GGCGGCATCTACAAGTTCAT	GGATCTTGAGGAAGCAGTCG
Tg	GTCCAATGCCAAAATGATGGTC	GAGAGCATCGGTGCTGTTAAT
Tshr	GTCTGCCCAATATTTCCAGGATCTA	GCTCTGTCAAGGCATCAGGGT
Tpo	ACAGTCACAGTTCTCCACGGATG	ATCTCTATTGTTGCACGCCCC
Duox2	AAGTTCAAGCAGTACAAGCGAT	TAGGCACGGTCTGCAAACAG
Duoxa2	GACGGGGTGCTACCCTTTTAC	GCTAAGAAGGACTCTCACCAAC
